# Health professionals’ views and experiences of breaking bad news in the Eastern Mediterranean Region: a scoping review

**DOI:** 10.3389/fmed.2024.1440867

**Published:** 2024-09-03

**Authors:** Abdulla Ahmad A. A. Yousuf, Derek Charles Stewart, Tanya Kane, Abderrezzaq Soltani, Abdullatif Al-Khal, Ahsan Sethi

**Affiliations:** ^1^QU Health, Qatar University, Doha, Qatar; ^2^Hamad Medical Corporation, Doha, Qatar

**Keywords:** truth disclosure, Qatar, Middle East, MENA, breaking bad news

## Abstract

**Introduction:**

Breaking bad news is a critical yet challenging aspect of healthcare that requires effective communication skills, empathy, and cultural sensitivity. Health professionals in the World Health Organization’s (WHO) Eastern Mediterranean Region face unique cultural and social factors distinct from other parts of the world. This scoping review aims to comprehensively explore the peer-reviewed literature on the health professionals’ experiences in delivering bad news within the WHO’s Eastern Mediterranean Region.

**Methods:**

This scoping review was conducted according to the Joanna Brigg Institute’s scoping review methodology and reported utilizing the Preferred Reporting Items for Systematic Reviews extension for scoping review (PRISMA-ScR) guidelines. A search using a combination of keywords and MeSH terms related to “breaking bad news” and “health professionals” was performed in PubMed, Scopus, CINAHL, EBSCO, ERIC via Embase, and Dar Almandumah (Arabic) databases. Common themes were synthesized from studies conducted in the WHO’s Eastern Mediterranean Region.

**Results:**

Out of 4,883 studies initially identified in the databases, 24 studies met the inclusion criteria, involving a total of 4,710 participants, including physicians, nurses, and residents. The studies were published between 2006 and 2022, predominantly from Iran (*n* = 12). The majority employed a cross-sectional design (*n* = 21) or mixed methods (*n* = 3), with a notable absence of qualitative studies. No studies used theoretical frameworks. More than half of the studies (*n* = 14) reported that participants had positive attitudes toward breaking bad news. This positivity was evident in their willingness to share bad news, perceived possession of adequate knowledge, positive attitudes, having received training, awareness of accepted approaches, and adherence to protocols. The lack of training and limited awareness of established protocols like SPIKES, ABCDE, and BREAKS for breaking bad news were major concerns among participants.

**Conclusion:**

The scoping review reveals both positive and negative experiences of breaking bad news by health professionals in the WHO’s Eastern Mediterranean Region. Most studies highlight the need for culturally sensitive targeted education and training programs on breaking bad news. Further research, particularly using qualitative methodologies and theoretical frameworks is warranted.

## 1 Introduction

Breaking bad news to patients is widely recognized as a key aspect of clinical practice, yet it remains challenging for health professionals at all levels of experience (1–4). Acknowledging that the interpretation of what may be considered neutral, good, or bad is subjective, Ptacek and Eberhardt define “bad news” as, information that results in a cognitive, behavioral, and/or emotional deficit in the person receiving the news, which persists for a while (5). An individual’s response may be shaped by one’s life experiences, personality, spiritual beliefs, philosophical stance, perceived social support and/or emotional resilience (1, 6).

Breaking bad news can have devastating effects on patients and their families. Such disclosures may generate feelings of hopelessness and potentially adversely impact patient outcomes (7, 8). In addition, the manner in which bad news is delivered can exacerbate a patient’s emotional distress (9), and influence a patient’s perception of their condition and their adherence to the management plan (10).

For many health professionals, breaking bad news can be a daunting task. Evidence links this responsibility to exhaustion, fatigue, and burnout (4, 11–14); fear of making diagnostic errors and self-blame (12); poor practitioner self-care (12); and a reduced sense of personal accomplishment (13). Novice health professionals, in particular, may lack the experience to fully comprehend the myriad of patients’ concerns, and address these issues inadequately, fostering feelings of mistrust, anger, and fear (15). The challenges of breaking bad news may be compounded by factors beyond the patient-professional relationship, including family involvement, cultural influences, and institutional constraints (16).

Education and training at undergraduate and postgraduate levels, complemented with ongoing professional development, are essential to equip health professionals to break bad news effectively. Related interventions are associated with significant improvements in observer-rated news delivery skills and moderate improvements in confidence (17). Indeed, those who have not received formal education and training in this area often feel ill-prepared for the task (18). Although breaking bad news is recognized as an essential skill in undergraduate and graduate health programs (19), many health professionals report feeling ill-equipped and express the need for additional training (20, 21).

Protocols like SPIKES (Setting up, Perception, Invitation, Knowledge, Emotions with Empathy, and Strategy or Summary) (22) and the ABCDE model (Advanced preparation, Building therapeutic relationship, Communicating effectively, Dealing with reactions, and Encouraging emotions) (23, 24) are increasingly employed to provide a structure to breaking bad news with synthesized evidence of their effectiveness (25). Respect, support, and empathy are central to these approaches which aim to mitigate the negative impact of breaking bad news (26).

Effective communication of bad news has been studied extensively in Western medical contexts, which often emphasize secularism, individualism, and patient autonomy (27). In Western culture, patients expect to be provided with at least some information about their disease and an estimate of their prognosis (28). The gold standard for delivering bad news in the West is to speak directly to the patient and their family (29). Western ethics unequivocally supports disclosing bad news to patients. Concealing the truth can lead to a crisis of conscience and psychological exhaustion among healthcare workers (30). However, it is important to acknowledge that the Western emphasis on autonomy and truth is distinctive and not universally shared, with some cultures prioritizing harmony over truth (31). These cultural differences can lead to professional dilemmas. For instance, Malaysian medical students studying a Western curriculum noted a significant disparity: 64% of students in Malaysia reported that relatives are informed of a diagnosis before the patient, compared to only 2% of students in the UK (32). In contrast, in the UK, withholding a diagnosis from the patient would violate professional guidelines and would only be permissible within the legal framework in exceptional circumstances (33, 34).

Less is known, however, about disclosures of bad news in non-Western cultural milieus, such as the World Health Organization (WHO) Eastern Mediterranean Region (EMR) (28). This predominantly Arab-Muslim region comprises 21 countries with a population of 679 million (35). Healthcare professionals in this region navigate a complex landscape of social, religious, and linguistic factors when breaking bad news. The religious and paternalistic cultural values guiding behavior, decision-making, perception, delivery, and the experiences of the stakeholders in the EMR need examination (36–39). This scoping review aims to address this gap by synthesizing the peer-reviewed literature on health professionals’ experiences of breaking bad news within the Eastern Mediterranean region. The findings of this scoping review will help understand cultural aspects and practices regarding breaking bad news in the EMR.

## 2 Methods

The scoping review was conducted in accordance with the Joanna Brigg Institute’s (JBI) methodology for scoping reviews (40) and is reported following the Preferred Reporting Items for Systematic Reviews extension for scoping review (PRISMA-ScR) guidelines (41).

The study inclusion criteria were based on the population, concept, and context model. Studies reporting views and experiences of any health professional population were included, with “breaking bad news” as the review concept. The geographic context was the WHO Eastern Mediterranean Region, which includes the Gulf Cooperation Council (GCC) members (Qatar, Saudi Arabia, Bahrain, Oman, Kuwait, and the United Arab Emirates), Djibouti, Egypt, Iran, Iraq, Israel, Jordan, Libya, Lebanon, Morocco, Syria, Malta, Tunisia, West Bank and Gaza, and Yemen (35).

The search was conducted in PubMed, Scopus, Embase, Cumulative Index to Nursing and Allied Health Literature (CINAHL), EBSCO, Education Resources Information Center (ERIC), and Dar Almandumah (Arabic database) to identify the relevant literature. The key search terms were “breaking bad news,” “health professionals,” and “Eastern Mediterranean Region,” modified as necessary for each database (see Supplementary material 1). Databases were searched from inception until July 2023. No language limitations were applied, ensuring a comprehensive review across diverse linguistic sources such as English and Arabic. Review articles, letters, opinion papers, and editorials were excluded. All identified citations were collated and uploaded into EndNote Web (Clarivate Analytics, PA, USA) and duplicates were removed. The remaining citations were exported to Rayyan QCRI® (42). Two independent reviewers screened titles and abstracts followed by full text for eligibility, with disagreements resolved by discussion or consultation with a third reviewer.

Data extraction was conducted using a pre-piloted Microsoft Excel® spreadsheet. Extracted data included authors, year of publication, title, journal, country, study aim, design, professions of participants, number of participants (response rate), setting, method, any theory used, data collection tool development and validation, key findings, stated study strengths and weaknesses, and conclusion. As with the screening phase, data extraction was performed by two independent reviewers, with disagreements resolved by a third reviewer. Data synthesis was then conducted using a narrative approach to identify key themes and patterns in the data related to the review aim. Similarly, each article was reviewed by two independent reviewers to identify the key patterns and themes during data synthesis. However, five reviewers were involved in the process, with one reviewer reviewing all articles and the other four equally reviewing all articles. Disagreements were resolved with mutual discussion. Data was coded manually by each independent reviewer.

## 3 Results

**Figure 1:**
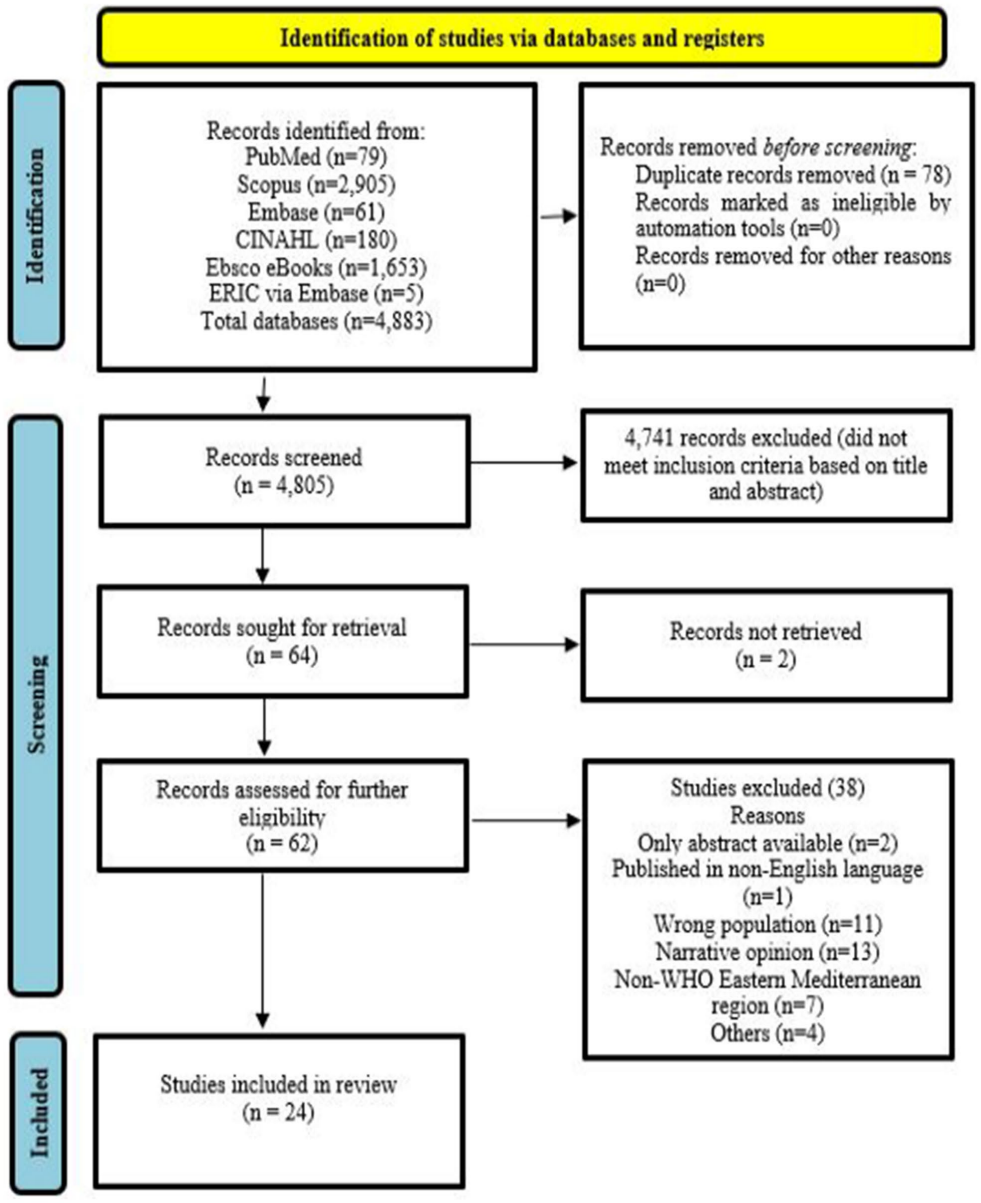
PRISMA flow diagram for the scoping review.

The search yielded 4,883 articles, of which 4,805 remained after removing duplicates. Dar Almandumah (Arabic database) produced no results. Title and abstract screening reduced the number to 64, with 24 retained for data extraction and mapping (Figure 1).

Study characteristics including aims, countries, study designs, participants, setting, use of theory, and data collection tool development are provided in Table 1.

**Table 1:** Data extraction of study characteristics.

Authors	Year	Stated aim	Country	Design	Participants, n (response rate)	Specialty and experience	Setting	Theory	Data collection tool development
Amiel et al. (43)	2006	Evaluate the reliability and validity of a competence-based assessment, utilizing simulated patients as evaluators, to assess primary care physician’s ability to deliver bad news	Not stated but all authors were from Israel hence assumed from Israel	Non-randomized controlled study	34 general practitioners; 17 in the study group, 17 in the control	-	General practice	None	Based on “How to Break Bad News” by Buckman. Trained simulated patients presented the scenarios in 8 stations who evaluated candidates utilizing global ratings of 2 Likert scale questionnaires.
Arbabi et al. (38)	2010	Assessment of attitude towards breaking bad news to patients	Iran	Cross-sectional survey	50 physicians and 50 nurses	-	Cancer Institute	None	Questionnaire based on literature review. Focused on patients’ and doctors’ interviews, and the factors affecting how to disclose diagnosis and bad news. Content validity assessed by 5 oncology and psychiatry professors. No details of piloting.
Al-Mohaimeed and Sharaf (44)	2013	Explore the perspective and practices regarding breaking bad news to patients	Saudi Arabia	Cross-sectional survey	458 physicians (30%)	GP/Family Medicine (*n* = 184, 40.2%), Pediatrics/ Medicine (*n* = 53, 11.6%), Surgery (*n* = 38, 8.3%), OB/Gynae (*n* = 48, 10.5%), Psychiatry (*n* = 10, 2.2%), Others (*n* = 125, 27.3%)	Public and private hospitals	None	Developed from SPIKES protocol. Questionnaire validated by 3 experts in communication skills. No details of piloting.
Shomoossi et al. (45)	2013	Investigate the delivery of death notifications by nurses	Iran	Cross-sectional survey	97 nurses (response rate not given)	-	Hospital	None	Questionnaire developed from review of published literature and ABCDE strategies. Assessed test–retest and validity co-efficient. No details of piloting.
Naji et al. (46)	2014	Examine disclosure practices and factors affecting them	Lebanon	Cross-sectional survey	500 physicians (69%)	-	Hospital	None	Questionnaire based on previous study. No details of validity testing or piloting.
Farhat et al. (47)	2015	Identify the attitudes regarding the disclosure of a cancer diagnosis	Lebanon	Cross-sectional survey	363 patients, families, friends, nurses, and physicians (94.5%). 13% of respondents were oncologists and other specialists	-	Hospital	None	Questionnaire based on previous study. No details given of further validity testing or piloting.
Imanipour et al. (48)	2015	Determine the role, perspective, and knowledge regarding breaking bad news	Iran	Cross-sectional survey	160 nurses (response rate not given)	ICU 141 (88.1%), CCU 19 (11.9%); Experience (years): 1–5 (57.5%), 5–10 (26.9%), 10–15 (8.1%), 15–20 (5.6%), >20 (1.9%)	ICU	None	Questionnaire based on the SPIKES protocol. Content validity assessed by professors in medical ethics, psychiatry, and nursing. Pilot study conducted, test–retest reliability.
Ozyemisci-Taskiran et al. (49)	2016	Explore experiences and opinions about breaking bad news to patients with spinal cord injury	Turkey	Cross-sectional survey	69 physiatrists (response rate not given)	Residents (*n* = 32), clinical experience = 3 (1–8) years, Specialists (*n* = 37), clinical experience = 12 (3–41) years	Hospital	None	Questionnaire based on the SPIKES protocol, literature and interviews with experts. No details given of further validity testing or piloting.
Adeli et al. (50)	2016	Examine the attitudes regarding revealing influential news to patients	Iran	Cross-sectional survey	150 physicians (100%)	-	Public, private sector or both	None	Questionnaire based on expert panel recommendations. Face validity by faculty members and internal reliability tested. No details given of piloting.
Borgan et al. (51)	2018	Assess the truth disclosure practices when encountering patients with serious illness	Jordan	Cross-sectional survey	240 physicians (60.8%)	General Practitioner (*n* = 31, 19%), Resident (*n* = 95, 58%), Specialist (*n* = 38, 23%)	4 Hospitals	None	Questionnaire based on previous study. No details of validity testing. Piloted in 15 physicians.
Muneer et al. (52)	2018	Assess the attitude and practice regarding breaking bad news	Sudan	Cross-sectional survey	291 physicians (54%)	Internal medicine 102 (64.2%), General surgery 57 (35.4%)	Teaching hospital	None	Questionnaire based on previous study. No details given of further validity testing or piloting.
Biazar et al. (53)	2019	Investigate the way bad news is delivered	Iran	Cross-sectional survey	243 specialists and residents (97%)	-	Hospital	None	Questionnaire based on previous study. Tested for validity and reliability. No details given of piloting.
Mostafavian and Shaye (39)	2018	Evaluate the ability and skills of physicians in delivering bad news to cancer patients	Iran	Cross-sectional survey	70 physicians (response rate not given)	Internal medicine 13 (18.6%), Surgery 16 (22.9%), Oncology 12 (17.1%), Gynecology 9 (12.9%), Urology 8 (11.4%), Dermatology 3 (4.3%), Pediatric 3 (4.3%), Neonatology 1 (1.4%), Neural surgery 4 (5.7%), Endocrinology 1 (1.4%)	2 hospitals	None	Questionnaire based on SPIKES protocol. No details given of piloting.
Tehran et al. (54)	2019	Evaluate the skill of general physicians in breaking bad news	Iran	Cross-sectional survey	200 general physicians (response rate not given)	-	Educational Hospital	None	Questionnaire based on SPIKES protocol. No detail given of piloting.
Shahi et al. (55)	2020	Assess physicians’ performance as well as the importance of their training on how to deliver bad news to patients diagnosed with cancer	Iran	Cross-sectional survey	12 physicians (100%)	-	Hospital	None	Questionnaire based on SPIKES protocol. No detail given of piloting.
Dafallah et al. (56)	2020	Assess adherence to the SPIKES protocol in breaking bad news	Sudan	Cross-sectional survey	192 doctors (100%)	Medicine (*n* = 39, 20.3%), General surgery (*n* = 25, 13%), Obstetrics and gynecology (*n* = 32, 16.7%), Pediatric (*n* = 22, 11.5%), Pediatric surgery (*n* = 11, 5.7%), Orthopedic (*n* = 23, 12%), Urology (*n* = 11, 5.7%), Nephrology (*n* = 10, 5.2%), Oncology (*n* = 11, 5.2%), ENT (*n* = 8, 4.2%)	Teaching Hospital	None	Questionnaire-based on SPIKES protocol. No details given of piloting.
Yazdanparast et al. (57)	2021	Evaluate the effect of communication skills training on the level of skill and participation of nurses in breaking bad news	Iran	Semi-experimental study	60 nurses (100%)	-	Educational Hospital	None	Questionnaire-based on SPIKES protocol. Tested for internal reliability. No details given of piloting.
Rezayof et al. (58)	2022	Design and evaluate a novel virtual instructional design for improving obstetrics and gynecology (OB/GYN) residents’ breaking bad news skills	Iran	Virtual instructional design	33 residents (Response rate not given)	Obstetrics and gynecology (OB/GYN)	Hospital	None	Questionnaire based on the ADDIE model (Analysis, Design, Development, Implementation and Evaluation). Content preparation included virtual training package multimedia, text, educational slides and videos.
AlZayani et al. (59)	2022	Assess attitudes and practices regarding truth-telling to seriously ill patients	Bahrain	Cross-sectional survey	156 residents and specialist physicians (72%)	Resident 24.1% (*n* = 27), Chief resident 1.8% (*n* = 2), Specialist 42.9% (*n* = 48), Consultant 31.3% (*n* = 35)	Public hospital	None	Questionnaire based on previous study. No details given of further validity testing or piloting.
Awny et al. (60)	2022	Explore knowledge, attitude, and practice toward palliative care	Egypt	Cross-sectional survey	220 physicians (response rate not given)	Resident (*n* = 52, 23.6%), Assistant lecturer (*n* = 101, 45.9%), Lecturer (*n* = 48, 21.8%), Assistant professor (*n* = 12, 5.5%), Professor (*n* = 7, 3.2%)	University Hospital	None	Questionnaire based on previous study. Validated by expert physicians. No details given of piloting.
Bazrafshan et al. (61)	2022	Identify the attitudes towards breaking bad medical news to patients	Iran	Cross-sectional survey	133 physicians (100%)	Internists (*n* = 81, 60.9%), Surgical specialists (*n* = 52, 39.09%)	Hospital	Not given	Questionnaire based on previous studies. Further validation by an expert panel. No details given of piloting.
Elashiry et al. (62)	2022	Assess knowledge, attitude, and practice regarding SPIKES protocol for breaking bad news	Egypt	Cross-sectional survey	395 physicians (response rate not given)	Surgery (*n* = 99, 25.1%), General Medicine (*n* = 74, 18.7%), Pediatrics (*n* = 63, 15.9%), GP and FP (*n* = 45, 11.4%), Obstetrics and Gynecology (*n* = 38, 9.6%), Oncology (Medical) (*n* = 20, 5.1%), Psychology (*n* = 12, 3.0%), Others (*n* = 44, 11.1%)	teaching Hospital	None	A questionnaire based on the SPIKES protocol and Santos et al., breaking bad news attitude scale used was validated by Santos et al. No details given of piloting.
Khalaf et al. (63)	2022	Assess the use of non-physical methods in breaking bad news	Iran	Cross-sectional survey	60 physicians (response rate not given)	Surgical (*n* = 21), Medical (*n* = 19), Emergency (*n* = 20)	Hospital	None	Bespoke questionnaire. No details given of further validity testing or piloting.
Rayan et al. (64)	2022	Examine critical care nurses’ attitudes, roles, experience, education, and barriers regarding breaking the bad news	Jordan	Cross-sectional survey	210 nurses working in ED, ICU, or CCU (response rate not given)	Paramedic and ER 67 (31.9%), ICU 108 (51.4%), CCU 35 (16.7%)	Hospital	None	Questionnaire-based on the previous study. No details given of further validity testing or piloting. Internal reliability confirmed.

Half of the studies were conducted in Iran (*n* = 12), with 2 studies each from Lebanon, Jordan, Egypt, and Sudan, and one each from Saudi Arabia, Bahrain, and Turkey (N.B. one study did not specify the region). Most studies (*n* = 21) were cross-sectional, with one non-randomized controlled study, one semi-experimental study, and one adopting a virtual instructional design. There were no qualitative studies exploring the phenomenon in-depth or using theoretical frameworks. Study participants included physicians (15 studies), nurses (4), physicians and nurses (2), physicians and residents (2), and residents only (1). Study settings spanned private/public hospitals and medical centers, private/public university hospitals, cancer centers, and intensive care departments.

A total of 4,710 participants were included in the 24 studies, with the largest study reporting data from 500 participants (a cross-sectional survey of physicians, with a response rate of 69%). The smallest study had only 12 participants (100% response rate). Cross-sectional survey studies reported response rates ranging from 30 to 100%. Nine studies employed the SPIKES protocol in questionnaire development, while another nine adopted questionnaires from previously published studies. In the remaining cross-sectional studies, little detail was provided on questionnaire development. No study reported the use of any theory in the development of data collection tools or data analysis and interpretation. Similarly, few studies provided details on questionnaire piloting prior to usage.

The key findings and conclusions derived from each study are given in Table 2. The most common findings were willingness to share bad news, inadequate training for breaking bad news, lack of formal education, experiences varying with participant demographics, and lack of awareness and adherence to SPIKES and ABCDE protocols.

**Table 2:** Data extraction of key study findings and conclusions.

Authors	Year	Key findings	Conclusions
Amiel et al. (43)	2006	GPs in intervention arm had significant post-test average grade compared to pre-test. There was minimal improvement in control group.	Breaking bad news training should be validated before application in a healthcare setting. SPs are reliable evaluators of breaking bad news training.
Arbabi et al. (38)	2010	A minority of respondents were trained to deliver bad news. Respondents preferred to deliver bad news to patients alone or in the presence of patients’ partners. A minority agreed that they would explain life expectancy to patients.	There is an increase in willingness to share bad news compared to the past. Physicians and nurses lack adequate skills to deliver bad news to patients.
Al-Mohaimeed and Sharaf (44)	2013	The majority of participants shared bad news with their patients. The majority preferred to deliver bad news to relatives rather than patients. Physicians who had higher qualifications were less skilled in breaking bad news.	The majority of physicians lacked adequate skills to deliver bad news to patients. There is a need for training in this specific aspect of health care.
Shomoossi et al. (45)	2013	The majority of nurses did not receive any training in breaking bad news to patients. Almost all were unfamiliar with SPIKES protocols and were not aware of ABCDE protocols. All agreed on adopting ABCDE strategies for delivering death notifications.	There is an urgent need for training nurses regarding communication skills. Special attention should be given to patients’ emotions.
Naji et al. (46)	2014	More than half of participants agreed to share full truth disclosure with patients. Most disclosers attributed their disclosure practices mostly to medical education and professional experience.	There is an increasing trend regarding willingness to disclose bad news to patients. Disclosure is likely to become a normative practice.
Farhat et al. (47)	2015	Three-quarters of physicians agreed that cancer diagnosis should be shared with patients. A minority revealed cancer diagnosis. Only a few would reveal the diagnosis immediately.	Physicians want to communicate the diagnosis of cancer. In practice, diagnosis revealed progressively over the course of treatment.
Imanipour et al. (48)	2015	The majority of respondents had a positive attitude towards the involvement of nurses in breaking bad news. Almost three quarters had moderate knowledge about breaking bad news to patients. A minority had good knowledge of breaking bad news.	Critical care nurses have a positive attitude towards breaking bad news. Critical care nurses have inadequate knowledge level regarding breaking bad news.
Ozyemisci-Taskiran et al. (49)	2016	Almost half of the respondents received basic communication skills training. All agreed that physiatrists should participate in breaking bad news to patients. More than half believed that the most appropriate time for relaying bad news to patients was during rehabilitation. A minority told the absolute truth to the patients.	There was a difference in the opinions regarding the style of delivering bad news to patients. There was a lack of satisfaction concerning communication skills. There is a need for the development of communication skills through training and intervention.
Adeli et al. (50)	2016	More than half of physicians revealed that they were forced to tell lies to patients. Almost half had an average attitude level regarding breaking bad news to patients. Male respondents demonstrated superior attitude levels compared to females. There was a positive relationship between work experience and attitude.	Most physicians think that withholding bad news from patients is not absolutely prohibited. While breaking bad news, knowledge, awareness, and age of patients should be kept in mind.
Borgan et al. (51)	2018	One quarter of the physicians did not share the bad news with their patients. The majority directly shared bad news with patients.	The majority of physicians shared bad news with their patients. In select cases, most will make exceptions.
Muneer et al. (52)	2018	Almost half of respondents received training regarding breaking bad news to patients. The majority thought that the patient should be told everything about his or her serious illness. If pressured by a relative to hide the truth, almost half agreed that they would break bad news if the patient was willing to listen. Only one quarter followed a standardized protocol for breaking bad news.	A minority of respondents did not follow the protocols, indicating a lack of knowledge. There is a need for training of healthcare professionals regarding breaking bad news.
Biazar et al. (53)	2019	Only a limited number of participants received training in delivering bad news. Only a minority believed that they had the ability to deliver bad news to patients. No differences were noted among physicians who received training and those who did not.	There is a need for training of the physicians to deliver bad news to patients.
Mostafavian and Shaye (39)	2018	All participants agreed not to tell bad news via telephone. The majority agreed to tell bad news in private. More than half believed that patient’s knowledge should be assessed before giving bad news.	Physicians do not have adequate knowledge about breaking bad news. There is a need to educate physicians regarding breaking bad news.
Tehran et al. (54)	2019	Most of the respondents did not receive any formal education relating to breaking bad news to the patients. Almost three quarters shared bad news with the patients, varying according to their experience. Half agreed that they considered patients’ fears while breaking bad news.	The skill level of the participants was desirable. There is a need for continuing education programs, especially for general physicians.
Shahi et al. (55)	2020	More males than females reported that patients have the right to know their diagnosis. More females than males had effective communication with patients when delivering bad news.	Guidelines can help physicians deliver bad news.
Dafallah et al. (56)	2020	Nearly half of physicians had experience in breaking bad news. More than half agreed that bad news should be delivered directly to the patients. The majority agreed that further training is needed in breaking bad news. Adherence to the SPIKES protocol was reported by more than half.	The majority of doctors adhere to the SPIKES protocol.
Yazdanparast et al. (57)	2021	There was significant improvement in breaking bad news related skills post intervention. Post intervention, there was significant increase in participation in delivering bad news to patients.	Communication skills are important for breaking bad news. The intervention could help health professionals in delivering breaking bad news.
Rezayof et al. (58)	2022	The majority of respondents believed that there was need for specific training, particularly in areas of interview context, strategy, planning, professionalism, empathy, knowledge, and receiving information.	Most obstetrics and gynecology residents do not have the necessary perceptions and skills to deliver bad news to patients.
AlZayani et al. (59)	2022	Almost half of respondents believed that patients should always be told about their diagnosis. One third did not know the breaking bad news policy of the hospital. The majority did not believe that withholding bad news from the patients was beneficial for them.	Physicians were not aware of any policies regarding breaking bad news in their hospitals.
Awny et al. (60)	2022	One third of respondents received education on palliative care. Around half preferred to break bad news and deliver prognosis to patients.	There is a need for training of the physicians on palliative care to the patients.
Bazrafshan et al. (61)	2022	The majority of physicians agreed on sharing bad news with patients. Most agreed that patients should be given bad news as soon as possible.	The desire to break bad news is lower compared to the tendency to hear bad news.
Elashiry et al. (62)	2022	Bad experiences of breaking bad news were reported by half of the physicians. Most physicians preferred breaking bad news to the patient’s family rather than the patient. Physicians’ agreement level with the SPIKES strategy was very high.	The majority of physicians highly agreed with the SPIKES strategy for breaking bad news, but they lacked essential knowledge. There is a need for further education and training regarding breaking bad news.
Khalaf et al. (63)	2022	The majority of the participants reported breaking bad news regularly. Less than half received training on breaking bad news. Only a minority received training on non-physical (in-person) breaking bad news.	A high proportion of physicians lack the necessary skills to break bad news, especially using non-physical ways during the pandemic. Further training of physicians is required.
Rayan et al. (64)	2022	Most critical care nurses contributed to breaking bad news and had positive attitudes regarding breaking bad news. The majority reported that they did not receive any specific training regarding breaking bad news. Nurses face various barriers when breaking bad news.	Administrators should promote the involvement of critical care nurses in breaking bad news and address the challenges in the process of breaking bad news. Training courses should also be offered to improve nurses’ skills.

Mapping identified themes of

- Positive views and experiences
- Negative views and experiences
- Practice variation with demographics and experience
- Need for education or further training

Table 3 provides the synthesis relating to the mapping of each study to these themes, which are described in further detail.

**Table 3:** Synthesis mapping of key study findings, identifying positive and negative views and experience.

Authors	Year	Positive views and experiences					Negative views and experiences					
		Perceived adequate Knowledge/ skills	Positive attitude towards breaking bad news	Received training	Awareness of accepted approaches (e.g., SPIKES/ABCDE)	Adherence to accepted approaches	Reported lack of training	Unaware of accepted approaches	Lack of full disclosure to patients	Unaware of institutional policy	Practice variation with demographics and experience	Need for education/training
Amiel et al. (43)	2006			✓								
Arbabi et al. (38)	2010		✓				✓		✓			✓
Al-Mohaimeed and Sharaf (44)	2013								✓		✓	✓
Shomoossi et al. (45)	2013		✓				✓	✓				✓
Naji et al. (46)	2014	✓	✓								✓	
Farhat et al. (47)	2015		✓						✓			
Imanipour et al. (48)	2015	✓	✓									✓
Ozyemisci-Taskiran et al. (49)	2016		✓	✓					✓			✓
Adeli et al. (50)	2016								✓		✓	
Borgan et al. (51)	2018								✓			
Muneer et al. (52)	2018		✓	✓		✓			✓			✓
Biazar et al. (53)	2019						✓					✓
Mostafavian and Shaye (39)	2018						✓		✓			✓
Tehran et al. (54)	2019	✓					✓				✓	✓
Shahi et al. (55)	2020		✓								✓	✓
Dafallah et al. (56)	2020	✓				✓						✓
Yazdanparast et al. (57)	2021	✓		✓								✓
Rezayof et al. (58)	2022		✓				✓					✓
AlZayani et al. (59)	2022		✓							✓		
Awny et al. (60)	2022		✓				✓					✓
Bazrafshan et al. (61)	2022		✓									
Elashiry et al. (62)	2022				✓	✓			✓			✓
Khalaf et al. (63)	2022		✓				✓					✓
Rayan et al. (64)	2022		✓				✓					✓

### 3.1 Positive views and experience

There were five themes of positive views and experiences: perceived adequate knowledge/skills, positive attitude towards breaking bad news, having received training, awareness of accepted approaches (e.g., SPIKES/ABCDE), and adherence to accepted approaches.

#### 3.1.1 Perceived adequate knowledge/skills

Of the 24 studies reviewed, five reported positive aspects regarding perceived knowledge and skills being adequate. These studies were all cross-sectional, with the participants in three studies being nurses and in two being physicians. Naji et al. (46) reported an association between higher perceived knowledge/skills and younger physicians with a high number of weekly practice hours.

#### 3.1.2 Positive attitude towards breaking bad news

Most studies (*n* = 14) reported participants’ positive attitudes regarding breaking bad news. Thirteen studies were cross-sectional, and one reported the development of a virtual instructional medium. Six of the studies included physicians, three included nurses, two each included physicians/nurses and physicians/residents, and one with residents only. Arbabi et al. (38) found an association between older age and experience and positive attitudes towards breaking bad news.

#### 3.1.3 Received training

Four studies reported that participants had received formal training in breaking bad news. Two had a cross-sectional design, one a semi-experimental study, and one a non-randomized controlled study design. All studies involved physicians, with one also including nurses. The percentage of participants receiving training on breaking bad news ranged from 15.9 to 50.9%.

#### 3.1.4 Awareness of accepted approaches (e.g., SPIKES/ABCDE)

Only one cross-sectional study of physicians explicitly reported that participants were aware of SPIKES/ABCDE approaches. In this study, Elashiry et al. (62) reported that 10% were aware of the SPIKES protocol. Dafallah et al. (56) and Muneer et al. (52) reported adherence to SPIKES/ABCDE protocols but did not assess the level of awareness among their participants.

#### 3.1.5 Adherence to accepted approaches

Three studies reported that participants adhered to accepted protocols while breaking bad news. All were cross-sectional studies of physicians. Elashiry et al. (62) reported that 91.8% of physicians agreed with the SPIKES protocol. They further noted that adherence level was significantly higher among male participants and those who received training about breaking bad news. Dafallah et al. (56) reported adherence to the SPIKES protocol ranging from 35 to 79%. According to Muneer et al. (52), only 55.6% of participants followed the SPIKES protocol, with others following BREAKS (Background, Rapport, Explore, Announce, Kindling, Summarise; 25%) and ABCDE (11.1%).

### 3.2 Negative views and experiences

There were five themes of negative views and experiences: reported lack of training, unawareness of accepted approaches, lack of full disclosure to patients, unawareness of institutional policy, and practice variation with demographics and experience.

#### 3.2.1 Reported lack of training

In nine studies, the respondents explicitly reported a lack of training in delivering bad news. These studies were largely cross-sectional, with five studies including physicians, two including physicians/residents, one including nurses, and one including both physicians and nurses. The remaining studies did not report any aspect of training.

#### 3.2.2 Unaware of accepted approaches

One study reported that participants were unaware of the accepted approaches for breaking bad news. This cross-sectional study on nurses by Shomoossi et al. (45) reported that almost all participants were unaware of SPIKES and ABCDE protocols.

#### 3.2.3 Lack of full disclosure to patients

Nine studies reported that participants did not disclose bad news in full. These cross-sectional studies were largely of physicians, with one study including nurses. Arbabi et al. (38) reported that a minority of physicians always discussed patients’ diagnoses, compared to two-thirds of nurses. Moreover, nurses mostly communicated with families rather than patients. Similarly, Al-Mohaimeed and Sharaf (44) reported that almost three-quarters of respondents delivered bad news to relatives rather than patients. Farhat et al. (47), also reported that a minority of physicians broke bad news to their patients. Only one-fifth of the respondents in a study by Ozyemisci-Taskiran et al. (49) told the “absolute truth” to patients, while the remainder conveyed a “partial truth”. Borgan et al. (51), and Muneer et al. (52) reported that most physicians complied with family requests for non-disclosure of bad news to patients. According to Elashiry et al. (62), most physicians preferred discussing bad news with family members rather than directly with patients.

#### 3.2.4 Unaware of institutional policy

In a cross-sectional study, AlZayani et al. (59) reported that one-third of physicians and residents were not aware of the institutional policy on breaking bad news.

### 3.3 Practice variation with demographic characteristics

Five cross-sectional studies of physicians reported that breaking bad news practice varied based on participant characteristics and experience. For example, Al-Mohaimeed and Sharaf (44) demonstrated that primary healthcare physicians were more open when breaking bad news to patients. Naji et al. (46) reported that those who disclosed bad news were more involved in medical teaching compared to non-disclosers Tehran et al. (54) reported significant differences in practices among different age groups.

### 3.4 Need for education/training

Most studies (*n* = 17) emphasized the need for education and training in breaking bad news. Of these, 10 studies involved physicians, four involved nurses, two involved physicians/residents/nurses, and one involved residents only. Most studies (*n* = 15) were cross-sectional, with two being semi-experimental with a virtual instructional design.

## 4 Discussion

### 4.1 Key findings

Twenty-four studies were identified in this scoping review of the peer-reviewed literature on health professionals’ views and experiences of breaking bad news in the WHO Eastern Mediterranean region. The Dar Almandumah (Arabic database) reported no results. Half of these studies were from Iran and were cross-sectional, with no studies reporting the use of theory. Most studies included physicians with very few reporting data from nurses and none from other health professionals. Mapping of study results generated four major themes: positive views and experiences (perceived adequate knowledge/skills, positive attitude towards breaking bad news, received training, awareness of accepted approaches (e.g., SPIKES/ABCDE), adherence to accepted approaches); negative views and experiences (reported lack of training, unaware of accepted approaches, lack of full disclosure to patients, unaware of institutional policy); practice varying with demographics and experience; and the need for education/training.

### 4.2 Strengths and weaknesses

The JBI method for scoping reviews was adhered to throughout, and the review was reported according to the Preferred Reporting Items for Systematic Reviews and Meta-analyses extension (PRISMA-ScR). Despite searching in an Arabic database, all the papers included in the review were in English language. There was only one non-English study (Persian), which was excluded after screening. Despite a robust approach to the conduct and reporting of the review itself, the findings and conclusions are limited by the absence of qualitative studies and the absence of theory in data collection and analysis. Most studies provided limited details of the development of questionnaire domains and items. Response rates were also variable, with several studies not quantifying the response rate.

### 4.3 Interpretation

The findings of this scoping review align with existing literature on breaking bad news, emphasizing the global challenge of health professionals in effectively communicating bad news. Given that this is a key competency of clinical practice, it is evident that health professionals need appropriate education and training to develop and master this skill. Breaking bad news can have adverse consequences for patients and their families (7–10), and negatively impact those delivering the news (4, 11–16), underscoring the need for targeted education geared towards honing and enhancing these skills. Perhaps the most significant finding of this scoping review is healthcare providers’ articulation of the expressed need for specific training in this area. Of note were the reports of the absence of training, and a lack of awareness of accepted approaches and institutional policies pertaining to breaking bad news in this region. While several of these themes (i.e., perceived adequate knowledge and skills, having received training, awareness, and adherence to accepted protocols) were described as positive findings, they were only reported in a minority of studies.

These findings resonate with literature from other parts of the world, which report issues related to training and accepted models of practice (65). In a meta-synthesis of 40 studies, Bousquet et al. attributed the difficulty in breaking bad news to a lack of physician training (16). A similar finding was reported by Sharif et al. in an evidence synthesis of 14 studies of health professionals’ training in breaking bad news (14). There is convincing evidence that links training in breaking bad news to improvements in practice. A scoping review by Chow et al. reported that physicians who received training were more likely to experience personal accomplishment and less likely to feel emotional exhaustion and depersonalization (11). Similarly, a meta-analysis of 17 studies identified that training interventions were associated with large, significant improvements in observer-rated news delivery skills (17).

Despite the issues identified in the scoping review relating to specific aspects of training, the participants in most studies reported positive attitudes towards breaking bad news and accepted this as an important task. It is, therefore, of particular interest that many studies highlighted negative aspects in terms of the lack of full disclosure of bad news. In nine studies, the participants reported partial disclosure to patients or opted to discuss bad news with the families rather than patients themselves. While involving family members in the coping mechanism for patients receiving bad news is acknowledged (16), the findings of lack of full disclosure may reflect the cultural and religious context of the Eastern Mediterranean region. Unlike the Western emphasis on individualism, patient autonomy, full diagnosis disclosure, and litigiousness (27), there is a propensity within this region to conceal bad news in an effort to protect patients’ morale (66). Therefore, in some cases, cultural influences can take precedence over professional considerations. This can be the reason why in some studies physicians were willing to share the bad news with relatives rather than the patients (44). Another important aspect to consider in the Eastern Mediterranean region is the strong bonds between families and the patriarchic nature of families, with elders making the majority of the decisions without much consideration for an individual’s rights. This can significantly influence how bad news is delivered. Another aspect of this relationship is shared by Borgan et al. (51) who report that bad news carries a sense of dread and family, in a way, feels obligated to insulate the patient from such news. The discrepancy regarding gender roles in breaking bad news as reported by Elashiry et al. (62) can be explained by the fact that female doctors may avoid breaking bad news due to the fear of being harmed, while male doctors may be more respected in the community and thus are more courageous while breaking bad news. Due to these reasons, some authors advocate the use of the culture-based protocol for breaking bad news in each area, with physicians following their local guidelines (53).

There was a noticeable lack of the application of theory in all studies. The inclusion of theory (e.g., behavioral) in the development of data collection tools, data analysis, and interpretation enhances research robustness and rigor. Importantly, the use of behavioral theory allows consideration of all possible influences on and explanations for behavior and informs the design of effective interventions. The lack of theory in the studies may be one reason that few studies reported issues of negative impact on health professionals’ emotions, fatigue and burnout which have been reported in other related reviews (12, 13).

The studies captured were largely cross-sectional, which, while appropriate for quantifying views and experiences, lack the depth of data generated via qualitative research. It is also noticeable that half of the studies were from Iran with no uniform representation of the WHO Eastern Mediterranean region. Most studies reported the perspectives of physicians, with fewer nurses and none from other health professions. Given the multidisciplinary nature of healthcare and the increasingly clinical role of other health professionals, it is likely that the delivery of bad news may no longer be solely within the domain of physicians. Training and practice of other health professionals warrants further investigation.

The incorporation of structured training on this topic in health profession graduate programs globally is becoming increasingly recognized as essential to preparing the future workforce. For instance, in India, the AETCOM (Attitude, Ethics, and Communication) module is a comprehensive curriculum initiative aimed at enhancing medical students’ competencies in these areas (67). Similarly, in various other countries, there has been an increased emphasis on communication training in medical education (68, 69). For example, in Qatar, Hamad Medical Corporation has become mandatory for residents to complete their training (70). However, there is still a need for continuing education and training for breaking bad news.

### 4.4 Further research

Qualitative methods should be employed in future research to provide a deeper understanding of health professionals’ views and experiences when breaking bad news. Such qualitative studies should be designed with an underpinning theoretical framework and with greater consideration of the cultural factors influencing communication practices. There is also merit in conducting research that focuses on the development, implementation, and evaluation of novel approaches to health professional education and training in breaking bad news.

## 5 Conclusion

This scoping review provides a comprehensive mapping of existing literature on health professionals’ views and experiences of breaking bad news in the Eastern Mediterranean region. The findings revealed both positive and negative aspects while highlighting persistent challenges, with emphasis on the need for targeted education and training programs and the development of culturally sensitive communication protocols. Further research, particularly using qualitative methodologies, is warranted.

## Data Availability

The original contributions presented in the study are included in the article/Supplementary material, further inquiries can be directed to the corresponding author.
