# Supplementary material for: Health professionals’ views and experiences of breaking bad news in the Eastern Mediterranean Region: a scoping review
Source: Front Med (Lausanne). 2024 Sep 3;11:1440867. doi: 10.3389/fmed.2024.1440867 (PMC11407430; doi:10.3389/fmed.2024.1440867)
Supplement: Supplementary file 1 [file Table_1.docx]

Appendix A. Table containing results from all database search

| Key variables | Sub terms | Search options | SCOPUS | Embase | CINAHL | Ebsco eBooks | ERIC via Embase | PubMed |
| --- | --- | --- | --- | --- | --- | --- | --- | --- |
|  |  |  | Search Results  (Date: 6 July 2023) | Search Results  (Date: 6 July 2023) | Search Results  (Date: 6 July 2023) | Search Results  (Date: 6 July 2023) | Search Results  (Date: 6 July 2023) | Search Results  (Date: 6 July 2023) |
| 1. Breaking bad news | 1.1 Bad news | All fields | 127,727 | 3746 | 14703 | 2405 | 419 | 3288 |
| 2. Health Professional | 2.1 Health personnel | All fields | 722,974 | 98439 | 156302 | 773 | 13800 | 605376 |
|  | 2.2 Health professional* | TI OR AB | 264,184 | 89610 | 80845 | 3185 | 12642 | 67882 |
|  | 2.3 Healthcare professional* | TI OR AB | 78,014 | 53830 | 50316 | 1947 | 3423 | 36437 |
|  | 2.4 Doctor* | TI OR AB | 259,716 | 224738 | 74485 | 13978 | 16080 | 150300 |
|  | 2.5 Nurse* | TI OR AB | 407,380 | 397401 | 407282 | 7895 | 10231 | 318999 |
|  | 2.6 Radiologist* | TI OR AB | 83,270 | 99157 | 20691 | 427 | 54 | 61518 |
|  | 2.7 Pharmacist* | TI OR AB | 68,590 | 90970 | 24149 | 1319 | 520 | 42126 |
|  | 2.8 Dentist* | TI OR AB | 103,599 | 87477 | 24992 | 2230 | 1331 | 88829 |
|  | 2.9 Physiotherapist* | TI OR AB | 15,971 | 21700 | 9919 | 444 | 93 | 10554 |
|  | 2.10 Dietitian* | TI OR AB | 13,070 | 13172 | 6132 | 318 | 128 | 7765 |
|  | 2.11 Psychologist* | TI OR AB | 61,104 | 31533 | 10445 | 6788 | 10590 | 18794 |
|  | 2.12 Laboratory technician* | TI OR AB | 5,455 | 2283 | 453 | 257 | 425 | 1581 |
|  | 2.13 Physician* | TI OR AB | 560,495 | 672207 | 174891 | 6213 | 5609 | 454263 |
|  | 2.14 Allied health | TI OR AB | 16,134 | 15375 | 9053 | 624 | 1575 | 11936 |
|  | 2.15 Speech therapist* | TI OR AB | 6,687 | 2307 | 1902 | 428 | 869 | 1119 |
|  | 2.16 Psychotherapist* | TI OR AB | 9,932 | 6131 | 2366 | 1917 | 422 | 3481 |
| 3. WHO Eastern Mediterranean Region | 3.1 Middle East | Mesh (MH) |  |  | 3378 |  |  | 159818 |
|  | 3.2 Middle East | All fields | 951,985 | 40648 | 129624 | 5846 | 7052 | 228122 |
|  | 3.3 Afghanistan | All fields | 101,663 | 11852 | 17981 | 3342 | 691 | 8923 |
|  | 3.4 Bahrain | All fields | 42,482 | 7986 | 3612 | 773 | 280 | 4269 |
|  | 3.5 Djibouti | All fields | 6,782 | 835 | 1258 | 401 | 18 | 587 |
|  | 3.6 Egypt | All fields | 740,758 | 207030 | 35680 | 5465 | 1680 | 132038 |
|  | 3.7 Iran | All fields | 1,295,010 | 346954 | 107815 | 4169 | 4353 | 250507 |
|  | 3.8 Iraq | All fields | 270,682 | 47723 | 23129 | 3805 | 921 | 21923 |
|  | 3.9 Jordan | All fields | 1,610,702 | 101676 | 59618 | 5883 | 4490 | 48438 |
|  | 3.10 Kuwait | All fields | 123,973 | 22448 | 8164 | 1484 | 675 | 14038 |
|  | 3.11 Lebanon | All fields | 154,951 | 64942 | 23204 | 2156 | 951 | 39948 |
|  | 3.12 Libya | All fields | 46,285 | 2569 | 2500 | 1591 | 203 | 3564 |
|  | 3.13 Morocco | All fields | 249,984 | 43653 | 7888 | 2324 | 761 | 21876 |
|  | 3.14 Oman | All fields | 163,755 | 16873 | 9479 | 1148 | 772 | 13460 |
|  | 3.15 Palestine | All fields | 100,563 | 7153 | 4380 | 2429 | 716 | 4697 |
|  | 3.16 Qatar | All fields | 93,121 | 27037 | 9625 | 983 | 470 | 19540 |
|  | 3.17 Saudi Arabia | All fields | 604,310 | 175859 | 35278 | 2265 | 3076 | 133544 |
|  | 3.18 Somalia | All fields | 32,202 | 3539 | 5199 | 1490 | 259 | 3007 |
|  | 3. 19 Sudan | All fields | 177,386 | 22215 | 10250 | 2353 | 508 | 15716 |
|  | 3.20 Syria | All fields | 82,044 | 4274 | 6161 | 2768 | 487 | 6211 |
|  | 3.21 Tunisia | All fields | 233,288 | 65532 | 7508 | 1560 | 473 | 30392 |
|  | 3.22 United Arab Emirates | All fields | 161,889 | 102580 | 9666 | 1019 | 1261 | 22051 |
|  | 3.23 Yemen | All fields | 52,345 | 6743 | 4066 | 1217 | 224 | 4756 |
| 1. Overall | 1+2+3 | | 2,905 | 61 | 437 | 1653 | 0 | 79 |

Search string for PubMed:

((Bad news) AND (((((((((((((((("Health Personnel"[Mesh]) OR (Health professional* [Title/Abstract])) OR (Healthcare professional*[Title/Abstract])) OR (Doctor*[Title/Abstract])) OR (Nurse*[Title/Abstract])) OR (Radiologist*[Title/Abstract])) OR (Pharmacist*[Title/Abstract])) OR (Dentist*[Title/Abstract])) OR (Psychotherapist*[Title/Abstract])) OR (Physiotherapist*[Title/Abstract])) OR (Dietitian*[Title/Abstract])) OR (Psychologist*[Title/Abstract])) OR (Laboratory technician*[Title/Abstract])) OR (Physician*[Title/Abstract])) OR (Allied health[Title/Abstract])) OR (Speech therapist*[Title/Abstract]))) AND ((((((((((((((((((((((("Middle East"[Mesh]) OR (Middle East)) OR (Afghanistan)) OR (Bahrain)) OR (Djibouti)) OR (Egypt)) OR (Iran)) OR (iraq)) OR (Jordan)) OR (Kuwait)) OR (Lebanon)) OR (Libya)) OR (Morocco)) OR (Oman)) OR (Palestine)) OR (Qatar)) OR (Saudi Arabia)) OR (Somalia)) OR (Sudan)) OR (Syria)) OR (Tunisia)) OR (United Arab Emirates)) OR (Yemen))

Search string for Scopus:

( ( ALL ( middle AND east ) ) OR ( ALL ( afghanistan ) ) OR ( ALL ( bahrain ) ) OR ( ALL ( djibouti ) ) OR ( ALL ( egypt ) ) OR ( ALL ( iran ) ) OR ( ALL ( iraq ) ) OR ( ALL ( jordan ) ) OR ( ALL ( kuwait ) ) OR ( ALL ( lebanon ) ) OR ( ALL ( libya ) ) OR ( ALL ( morocco ) ) OR ( ALL ( oman ) ) OR ( ALL ( palestine ) ) OR ( ALL ( qatar ) ) OR ( ALL ( saudi AND arabia ) ) OR ( ALL ( somalia ) ) OR ( ALL ( sudan ) ) OR ( ALL ( syria ) ) OR ( ALL ( tunisia ) ) OR ( ALL ( united AND arab AND emirates ) ) OR ( ALL ( yemen ) ) ) AND ( ( ( TITLE ( psychotherapist* ) OR ABS ( psychotherapist* ) ) ) OR ( ( TITLE ( speech AND therapist* ) OR ABS ( speech AND therapist* ) ) ) OR ( ( TITLE ( allied AND health ) OR ABS ( allied AND health ) ) ) OR ( ( TITLE ( physician* ) OR ABS ( physician* ) ) ) OR ( ( TITLE ( laboratory AND technician* ) OR ABS ( laboratory AND technician* ) ) ) OR ( ( TITLE ( psychologist* ) OR ABS ( psychologist* ) ) ) OR ( ( TITLE ( dietitian* ) OR ABS ( dietitian* ) ) ) OR ( ( TITLE ( physiotherapist* ) OR ABS ( physiotherapist* ) ) ) OR ( ( TITLE ( dentist* ) OR ABS ( dentist* ) ) ) OR ( ( TITLE ( pharmacist* ) OR ABS ( pharmacist* ) ) ) OR ( ( TITLE ( radiologist* ) OR ABS ( radiologist* ) ) ) OR ( ( TITLE ( nurse* ) OR ABS ( nurse* ) ) ) OR ( ( TITLE ( doctor* ) OR ABS ( doctor* ) ) ) OR ( ( TITLE ( health AND professional* ) OR ABS ( health AND professional* ) ) ) OR ( ( TITLE ( healthcare AND professional* ) OR ABS ( healthcare AND professional* ) ) ) OR ( ALL ( health AND personnel ) ) ) AND ( ALL ( bad AND news ) )
